# 4-Methyl-6-phenyl­pyrimidin-2-amine

**DOI:** 10.1107/S1600536808008003

**Published:** 2008-03-29

**Authors:** Zhen-Jiang Li, Jun-E Huang, A-Lan Meng

**Affiliations:** a Qingdao University of Science and Technology, Qingdao 266061, People’s Republic of China

## Abstract

The title compound, C_11_H_11_N_3_, was synthesized as part of our research into functionalized pyrimidines. It crystallizes with two independent mol­ecules in the asymmetric unit that differ only in the twist between the two aromatic rings; the torsion angles between the rings are 29.9 (2) and 45.1 (2)°. The crystal packing is dominated by inter­molecular N—H⋯N hydrogen bonds between independent and equivalent mol­ecules, forming an infinite three-dimensional network.

## Related literature

For biological activity, see: Zhu & Yang (2005 ▶); Sherrington & Taskinen (2001 ▶); Ligthart *et al.* (2005 ▶). For a similar structure, see: Fun *et al.* (2006 ▶).
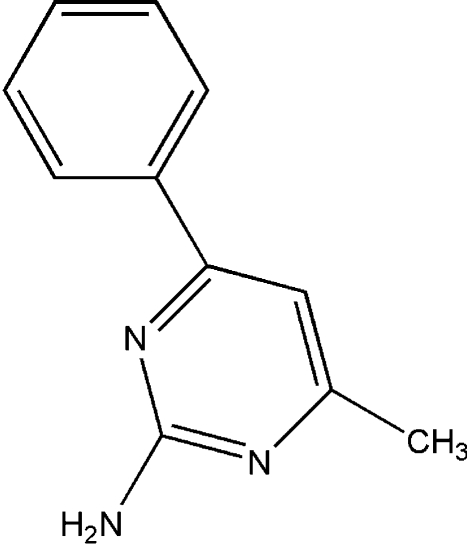


## Experimental

### 

#### Crystal data

C_11_H_11_N_3_
*M* *_r_* = 185.23
Monoclinic, 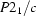

*a* = 14.0558 (11) Å
*b* = 9.3808 (7) Å
*c* = 18.5227 (12) Å
β = 125.950 (4)°
*V* = 1977.1 (2) Å^3^
*Z* = 8
Mo *K*α radiation
μ = 0.08 mm^−1^
*T* = 273 (2) K
0.30 × 0.20 × 0.20 mm

#### Data collection

Bruker SMART 1K CCD area-detector diffractometer
Absorption correction: multi-scan (*SADABS*; Sheldrick, 2004 ▶) *T*_min_ = 0.977, *T*_max_ = 0.985
17472 measured reflections
3619 independent reflections
2760 reflections with *I* > 2σ(*I*)
*R*_int_ = 0.033

#### Refinement

*R*[*F* ^2^ > 2σ(*F* ^2^)] = 0.039
*wR*(*F* ^2^) = 0.113
*S* = 1.04
3619 reflections
256 parameters
H-atom parameters constrained
Δρ_max_ = 0.21 e Å^−3^
Δρ_min_ = −0.16 e Å^−3^

### 

Data collection: *SMART* (Bruker, 2001 ▶); cell refinement: *SMART*; data reduction: *SAINT* (Bruker, 2001 ▶); program(s) used to solve structure: *SHELXTL* (Sheldrick, 2008 ▶); program(s) used to refine structure: *SHELXTL*; molecular graphics: *SHELXTL*; software used to prepare material for publication: *SHELXTL* and local programs.

## Supplementary Material

Crystal structure: contains datablocks h-1, I. DOI: 10.1107/S1600536808008003/fl2182sup1.cif

Structure factors: contains datablocks I. DOI: 10.1107/S1600536808008003/fl2182Isup2.hkl

Additional supplementary materials:  crystallographic information; 3D view; checkCIF report

## Figures and Tables

**Table 1:** Hydrogen-bond geometry (Å, °)

*D*—H⋯*A*	*D*—H	H⋯*A*	*D*⋯*A*	*D*—H⋯*A*
N1—H1*A*⋯N3^i^	0.86	2.38	3.1918 (18)	157
N1—H1*B*⋯N6^i^	0.86	2.35	3.2095 (18)	175
N4—H4*C*⋯N2^i^	0.86	2.29	3.1474 (19)	176
N4—H4*B*⋯N5^ii^	0.86	2.24	3.0834 (18)	166

Symmetry codes: (i) 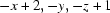; (ii) 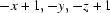.

**Fig. 1.**
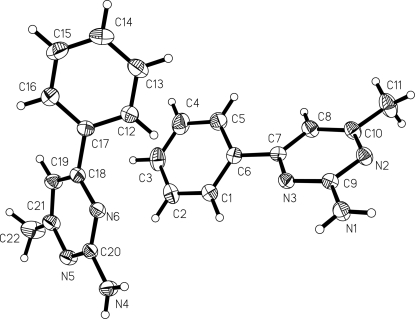
The molecular structure and atom-labeling scheme for (I), with displacement ellipsoids drawn at the 30% probability level.

**Fig. 2.**
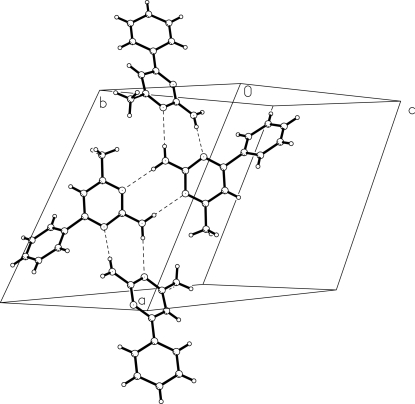
The packing of (I), showing one layer of molecules connected by N—H···N hydrogen bonds (dashed lines).

## supplementary crystallographic information

### Comment

Pyrimidines are broadly used in the preparation of pesticides and medications (Zhu & Yang, 2005). Functionalized pyrimidines are important for the synthesis of purine- and pteridine-related compounds and also for multiple hydrogen-bonding interactions that play a role in molecular recognition and supramolecular chemistry (Sherrington & Taskinen, 2001; Ligthart *et al.*, 2005). In the title compound, (I), (fig. 1) there are two molecules per asymmetric unit  that differ only in the twist between the two aromatic rings with dihedral angles between the phenyl and  pyrimidine rings of 29.41 (2)° 46.32 (3)°. Bond lengths and angles for (I) are generally normal (Fun *et al.*, 2006).

In the packing there are intermolecular N—H···N hydrogen bonds that link each independent molecule to related self molecules as well as to the second molecule in the asymmetric unit to create an infinite network of hydrogen bonded molecules  (Table 1, Fig. 2).

### Experimental

The single crystals of the title compound were obtained by reaction of 1-phenylbutane-1,3-dione(0.2 mmol) with guanidine nitrate(0.2 mmol) by refluxing in  DMF(50 ml). The product (yied 89%) was stirred in the DMF and single crystals of the title compound suitable for X-ray measurements were obtained by recrystallization from DMF at room temperature.

### Refinement

H atoms were fixed geometrically and allowed to ride on their attached atoms, with N—H=0.86, C—H=0.93 or 0.96 Å, and with *U*_iso_(H) values set at 1.5 *U*_eq_(C)(for CH3) or 1.2 *U*_eq_(C)(for CH2, aromatic CH and NH2).

### Crystal data

**Table:**

C_11_H_11_N_3_	*F*_000_ = 784
*M**_r_* = 185.23	*D*_x_ = 1.245 Mg m^−^^3^
Monoclinic, *P*2_1_/*c*	Mo *K*α radiation λ = 0.71073 Å
Hall symbol: -P 2ybc	Cell parameters from 512 reflections
*a* = 14.0558 (11) Å	θ = 2–22º
*b* = 9.3808 (7) Å	µ = 0.08 mm^−^^1^
*c* = 18.5227 (12) Å	*T* = 273 (2) K
β = 125.950 (4)º	Block, colorless
*V* = 1977.1 (2) Å^3^	0.30 × 0.20 × 0.20 mm
*Z* = 8	

### Data collection

**Table:**

Bruker SMART 1K CCD area-detector diffractometer	3619 independent reflections
Radiation source: fine-focus sealed tube	2760 reflections with *I* > 2σ(*I*)
Monochromator: graphite	*R*_int_ = 0.033
*T* = 295(2) K	θ_max_ = 25.4º
thin–slice ω scans	θ_min_ = 1.8º
Absorption correction: multi-scan(SADABS; Sheldrick, 2004)	*h* = −14→16
*T*_min_ = 0.977, *T*_max_ = 0.985	*k* = −11→11
17472 measured reflections	*l* = −22→20

### Refinement

**Table:**

Refinement on *F*^2^	Hydrogen site location: inferred from neighbouring sites
Least-squares matrix: full	H-atom parameters constrained
*R*[*F*^2^ > 2σ(*F*^2^)] = 0.039	*w* = 1/[σ^2^(*F*_o_^2^) + (0.0535*P*)^2^ + 0.393*P*] where *P* = (*F*_o_^2^ + 2*F*_c_^2^)/3
*wR*(*F*^2^) = 0.113	(Δ/σ)_max_ = 0.001
*S* = 1.04	Δρ_max_ = 0.21 e Å^−^^3^
3619 reflections	Δρ_min_ = −0.16 e Å^−^^3^
256 parameters	Extinction correction: SHELXL, Fc^*^=kFc[1+0.001xFc^2^λ^3^/sin(2θ)]^-1/4^
Primary atom site location: structure-invariant direct methods	Extinction coefficient: 0.0088 (13)
Secondary atom site location: difference Fourier map	

### Special details

**Table:**

Geometry. All e.s.d.'s (except the e.s.d. in the dihedral angle between two l.s. planes) are estimated using the full covariance matrix. The cell e.s.d.'s are taken into account individually in the estimation of e.s.d.'s in distances, angles and torsion angles; correlations between e.s.d.'s in cell parameters are only used when they are defined by crystal symmetry. An approximate (isotropic) treatment of cell e.s.d.'s is used for estimating e.s.d.'s involving l.s. planes.
Refinement. Refinement of *F*^2^ against ALL reflections. The weighted *R*-factor *wR* and goodness of fit *S* are based on *F*^2^, conventional *R*-factors *R* are based on *F*, with *F* set to zero for negative *F*^2^. The threshold expression of *F*^2^ > σ(*F*^2^) is used only for calculating *R*-factors(gt) *etc*. and is not relevant to the choice of reflections for refinement. *R*-factors based on *F*^2^ are statistically about twice as large as those based on *F*, and *R*- factors based on ALL data will be even larger.

### Fractional atomic coordinates and isotropic or equivalent isotropic displacement parameters (Å^2^)

**Table:**

	*x*	*y*	*z*	*U*_iso_*/*U*_eq_	
N1	1.09631 (12)	−0.10203 (14)	0.46842 (9)	0.0492 (4)	
H1A	1.0775	−0.1072	0.5049	0.059*	
H1B	1.1590	−0.1434	0.4810	0.059*	
N2	1.06247 (11)	−0.02652 (14)	0.33737 (9)	0.0445 (3)	
N3	0.93111 (10)	0.03294 (12)	0.37592 (8)	0.0371 (3)	
C1	0.69500 (14)	0.10273 (16)	0.31097 (10)	0.0428 (4)	
H1C	0.7276	0.0232	0.3478	0.051*	
C2	0.59049 (15)	0.1585 (2)	0.28979 (12)	0.0537 (4)	
H2B	0.5531	0.1161	0.3123	0.064*	
C3	0.54156 (17)	0.2762 (2)	0.23573 (13)	0.0661 (5)	
H3B	0.4718	0.3143	0.2223	0.079*	
C4	0.59617 (19)	0.3378 (2)	0.20139 (14)	0.0711 (6)	
H4A	0.5627	0.4168	0.1642	0.085*	
C5	0.70011 (16)	0.28266 (19)	0.22208 (12)	0.0557 (5)	
H5A	0.7363	0.3249	0.1986	0.067*	
C6	0.75185 (13)	0.16463 (15)	0.27758 (10)	0.0395 (4)	
C7	0.86232 (13)	0.10283 (14)	0.29811 (10)	0.0370 (3)	
C8	0.89113 (14)	0.11063 (17)	0.23828 (11)	0.0462 (4)	
H8A	0.8435	0.1598	0.1847	0.055*	
C9	1.02765 (13)	−0.02917 (15)	0.39158 (10)	0.0371 (3)	
C10	0.99219 (14)	0.04372 (18)	0.26026 (11)	0.0457 (4)	
C11	1.02670 (19)	0.0447 (3)	0.19755 (13)	0.0730 (6)	
H11A	1.1108	0.0476	0.2311	0.109*	
H11B	0.9937	0.1271	0.1597	0.109*	
H11C	0.9977	−0.0400	0.1616	0.109*	
N4	0.66133 (12)	0.04458 (13)	0.54000 (9)	0.0466 (4)	
H4B	0.6190	−0.0240	0.5377	0.056*	
H4C	0.7366	0.0363	0.5722	0.056*	
N5	0.49149 (11)	0.17062 (13)	0.44429 (9)	0.0440 (3)	
N6	0.68078 (11)	0.26712 (12)	0.49771 (8)	0.0379 (3)	
C12	0.78707 (15)	0.46852 (17)	0.44133 (12)	0.0494 (4)	
H12A	0.7997	0.3741	0.4339	0.059*	
C13	0.85244 (17)	0.5759 (2)	0.43871 (13)	0.0597 (5)	
H13A	0.9080	0.5533	0.4285	0.072*	
C14	0.83596 (17)	0.7156 (2)	0.45107 (13)	0.0644 (5)	
H14A	0.8809	0.7871	0.4499	0.077*	
C15	0.75362 (19)	0.74936 (19)	0.46501 (15)	0.0683 (6)	
H15A	0.7429	0.8438	0.4740	0.082*	
C16	0.68603 (17)	0.64335 (17)	0.46588 (13)	0.0566 (5)	
H16A	0.6286	0.6674	0.4738	0.068*	
C17	0.70284 (13)	0.50159 (15)	0.45505 (10)	0.0404 (4)	
C18	0.62748 (13)	0.38817 (15)	0.45327 (10)	0.0387 (4)	
C19	0.50733 (14)	0.40570 (17)	0.40489 (11)	0.0471 (4)	
H19A	0.4721	0.4919	0.3770	0.057*	
C20	0.60975 (13)	0.16496 (15)	0.49325 (10)	0.0376 (3)	
C21	0.44081 (14)	0.29142 (17)	0.39901 (11)	0.0475 (4)	
C22	0.30886 (16)	0.2934 (2)	0.33834 (15)	0.0744 (6)	
H22A	0.2787	0.2262	0.3593	0.112*	
H22B	0.2823	0.2681	0.2790	0.112*	
H22C	0.2812	0.3872	0.3379	0.112*	

### Atomic displacement parameters (Å^2^)

**Table:**

	*U*^11^	*U*^22^	*U*^33^	*U*^12^	*U*^13^	*U*^23^
N1	0.0443 (8)	0.0635 (8)	0.0430 (8)	0.0156 (6)	0.0274 (7)	0.0176 (7)
N2	0.0398 (7)	0.0573 (8)	0.0382 (8)	0.0040 (6)	0.0239 (7)	0.0047 (6)
N3	0.0349 (7)	0.0422 (6)	0.0335 (7)	0.0002 (5)	0.0196 (6)	0.0031 (5)
C1	0.0399 (9)	0.0490 (8)	0.0359 (9)	−0.0024 (7)	0.0202 (8)	−0.0030 (7)
C2	0.0455 (10)	0.0717 (11)	0.0479 (10)	−0.0032 (9)	0.0296 (9)	−0.0102 (9)
C3	0.0497 (11)	0.0876 (14)	0.0552 (12)	0.0223 (10)	0.0275 (10)	0.0005 (10)
C4	0.0712 (13)	0.0751 (13)	0.0652 (13)	0.0330 (11)	0.0391 (12)	0.0220 (10)
C5	0.0577 (11)	0.0579 (10)	0.0533 (11)	0.0135 (8)	0.0335 (10)	0.0141 (8)
C6	0.0377 (8)	0.0430 (8)	0.0328 (8)	0.0007 (6)	0.0179 (7)	−0.0014 (6)
C7	0.0351 (8)	0.0379 (7)	0.0334 (8)	−0.0026 (6)	0.0175 (7)	0.0012 (6)
C8	0.0432 (9)	0.0582 (9)	0.0353 (9)	0.0052 (7)	0.0220 (8)	0.0109 (7)
C9	0.0345 (8)	0.0403 (7)	0.0341 (8)	−0.0017 (6)	0.0187 (7)	0.0013 (6)
C10	0.0414 (9)	0.0617 (10)	0.0351 (9)	0.0007 (7)	0.0230 (8)	0.0039 (7)
C11	0.0642 (13)	0.1156 (17)	0.0521 (12)	0.0198 (12)	0.0414 (11)	0.0190 (11)
N4	0.0396 (7)	0.0426 (7)	0.0574 (9)	0.0040 (6)	0.0284 (7)	0.0128 (6)
N5	0.0377 (7)	0.0477 (7)	0.0461 (8)	0.0007 (6)	0.0243 (7)	0.0095 (6)
N6	0.0385 (7)	0.0396 (6)	0.0385 (7)	−0.0002 (5)	0.0242 (6)	0.0026 (5)
C12	0.0520 (10)	0.0467 (9)	0.0570 (11)	0.0015 (7)	0.0363 (9)	0.0049 (7)
C13	0.0559 (11)	0.0681 (11)	0.0666 (13)	−0.0038 (9)	0.0425 (10)	0.0081 (9)
C14	0.0619 (12)	0.0561 (11)	0.0710 (13)	−0.0176 (9)	0.0367 (11)	0.0032 (9)
C15	0.0769 (14)	0.0424 (9)	0.0895 (16)	−0.0120 (9)	0.0510 (13)	−0.0080 (9)
C16	0.0621 (11)	0.0473 (9)	0.0710 (12)	−0.0024 (8)	0.0449 (10)	−0.0038 (8)
C17	0.0416 (8)	0.0405 (8)	0.0381 (9)	−0.0007 (6)	0.0229 (7)	0.0038 (6)
C18	0.0429 (9)	0.0397 (8)	0.0378 (9)	0.0014 (6)	0.0260 (8)	0.0023 (6)
C19	0.0448 (10)	0.0448 (8)	0.0519 (10)	0.0071 (7)	0.0286 (9)	0.0139 (7)
C20	0.0391 (8)	0.0406 (7)	0.0366 (8)	0.0011 (6)	0.0243 (7)	0.0016 (6)
C21	0.0399 (9)	0.0543 (9)	0.0481 (10)	0.0049 (7)	0.0258 (8)	0.0125 (8)
C22	0.0415 (10)	0.0858 (14)	0.0792 (15)	0.0059 (10)	0.0261 (11)	0.0336 (12)

### Geometric parameters (Å, °)

**Table:**

N1—C9	1.3459 (19)	N4—C20	1.3467 (19)
N1—H1A	0.8600	N4—H4B	0.8600
N1—H1B	0.8600	N4—H4C	0.8600
N2—C10	1.339 (2)	N5—C21	1.339 (2)
N2—C9	1.3509 (19)	N5—C20	1.3484 (19)
N3—C9	1.3426 (19)	N6—C18	1.3431 (18)
N3—C7	1.3437 (18)	N6—C20	1.3510 (18)
C1—C2	1.382 (2)	C12—C13	1.383 (2)
C1—C6	1.392 (2)	C12—C17	1.384 (2)
C1—H1C	0.9300	C12—H12A	0.9300
C2—C3	1.374 (3)	C13—C14	1.373 (3)
C2—H2B	0.9300	C13—H13A	0.9300
C3—C4	1.379 (3)	C14—C15	1.363 (3)
C3—H3B	0.9300	C14—H14A	0.9300
C4—C5	1.375 (3)	C15—C16	1.382 (2)
C4—H4A	0.9300	C15—H15A	0.9300
C5—C6	1.391 (2)	C16—C17	1.386 (2)
C5—H5A	0.9300	C16—H16A	0.9300
C6—C7	1.483 (2)	C17—C18	1.488 (2)
C7—C8	1.387 (2)	C18—C19	1.380 (2)
C8—C10	1.378 (2)	C19—C21	1.384 (2)
C8—H8A	0.9300	C19—H19A	0.9300
C10—C11	1.499 (2)	C21—C22	1.502 (2)
C11—H11A	0.9600	C22—H22A	0.9600
C11—H11B	0.9600	C22—H22B	0.9600
C11—H11C	0.9600	C22—H22C	0.9600
			
C9—N1—H1A	120.0	C20—N4—H4B	120.0
C9—N1—H1B	120.0	C20—N4—H4C	120.0
H1A—N1—H1B	120.0	H4B—N4—H4C	120.0
C10—N2—C9	116.07 (13)	C21—N5—C20	116.55 (13)
C9—N3—C7	116.30 (12)	C18—N6—C20	115.84 (12)
C2—C1—C6	120.52 (15)	C13—C12—C17	120.02 (16)
C2—C1—H1C	119.7	C13—C12—H12A	120.0
C6—C1—H1C	119.7	C17—C12—H12A	120.0
C3—C2—C1	120.35 (17)	C14—C13—C12	120.58 (17)
C3—C2—H2B	119.8	C14—C13—H13A	119.7
C1—C2—H2B	119.8	C12—C13—H13A	119.7
C2—C3—C4	119.81 (17)	C15—C14—C13	119.89 (16)
C2—C3—H3B	120.1	C15—C14—H14A	120.1
C4—C3—H3B	120.1	C13—C14—H14A	120.1
C5—C4—C3	120.12 (18)	C14—C15—C16	120.07 (17)
C5—C4—H4A	119.9	C14—C15—H15A	120.0
C3—C4—H4A	119.9	C16—C15—H15A	120.0
C4—C5—C6	120.95 (18)	C15—C16—C17	120.81 (17)
C4—C5—H5A	119.5	C15—C16—H16A	119.6
C6—C5—H5A	119.5	C17—C16—H16A	119.6
C5—C6—C1	118.24 (15)	C12—C17—C16	118.59 (15)
C5—C6—C7	121.01 (14)	C12—C17—C18	120.67 (14)
C1—C6—C7	120.72 (13)	C16—C17—C18	120.65 (14)
N3—C7—C8	121.26 (14)	N6—C18—C19	121.89 (13)
N3—C7—C6	116.84 (13)	N6—C18—C17	117.21 (13)
C8—C7—C6	121.83 (14)	C19—C18—C17	120.86 (13)
C10—C8—C7	118.27 (14)	C18—C19—C21	118.12 (14)
C10—C8—H8A	120.9	C18—C19—H19A	120.9
C7—C8—H8A	120.9	C21—C19—H19A	120.9
N3—C9—N1	117.08 (13)	N4—C20—N5	116.82 (13)
N3—C9—N2	126.34 (13)	N4—C20—N6	117.13 (13)
N1—C9—N2	116.58 (13)	N5—C20—N6	126.02 (13)
N2—C10—C8	121.75 (14)	N5—C21—C19	121.31 (14)
N2—C10—C11	117.25 (15)	N5—C21—C22	116.64 (15)
C8—C10—C11	120.99 (15)	C19—C21—C22	121.98 (15)
C10—C11—H11A	109.5	C21—C22—H22A	109.5
C10—C11—H11B	109.5	C21—C22—H22B	109.5
H11A—C11—H11B	109.5	H22A—C22—H22B	109.5
C10—C11—H11C	109.5	C21—C22—H22C	109.5
H11A—C11—H11C	109.5	H22A—C22—H22C	109.5
H11B—C11—H11C	109.5	H22B—C22—H22C	109.5
			
C6—C1—C2—C3	−0.3 (2)	C17—C12—C13—C14	1.1 (3)
C1—C2—C3—C4	0.9 (3)	C12—C13—C14—C15	−0.8 (3)
C2—C3—C4—C5	−0.8 (3)	C13—C14—C15—C16	−0.6 (3)
C3—C4—C5—C6	0.0 (3)	C14—C15—C16—C17	1.7 (3)
C4—C5—C6—C1	0.7 (3)	C13—C12—C17—C16	0.0 (3)
C4—C5—C6—C7	178.44 (17)	C13—C12—C17—C18	176.76 (16)
C2—C1—C6—C5	−0.5 (2)	C15—C16—C17—C12	−1.4 (3)
C2—C1—C6—C7	−178.31 (14)	C15—C16—C17—C18	−178.14 (17)
C9—N3—C7—C8	−0.6 (2)	C20—N6—C18—C19	−1.1 (2)
C9—N3—C7—C6	176.50 (12)	C20—N6—C18—C17	−178.59 (13)
C5—C6—C7—N3	153.34 (15)	C12—C17—C18—N6	45.1 (2)
C1—C6—C7—N3	−28.9 (2)	C16—C17—C18—N6	−138.24 (16)
C5—C6—C7—C8	−29.6 (2)	C12—C17—C18—C19	−132.43 (17)
C1—C6—C7—C8	148.13 (15)	C16—C17—C18—C19	44.2 (2)
N3—C7—C8—C10	0.5 (2)	N6—C18—C19—C21	−3.4 (2)
C6—C7—C8—C10	−176.46 (14)	C17—C18—C19—C21	174.05 (15)
C7—N3—C9—N1	−178.71 (13)	C21—N5—C20—N4	179.01 (14)
C7—N3—C9—N2	0.8 (2)	C21—N5—C20—N6	−2.9 (2)
C10—N2—C9—N3	−0.9 (2)	C18—N6—C20—N4	−177.49 (13)
C10—N2—C9—N1	178.64 (14)	C18—N6—C20—N5	4.5 (2)
C9—N2—C10—C8	0.7 (2)	C20—N5—C21—C19	−2.0 (2)
C9—N2—C10—C11	−178.21 (16)	C20—N5—C21—C22	174.92 (16)
C7—C8—C10—N2	−0.6 (2)	C18—C19—C21—N5	5.0 (3)
C7—C8—C10—C11	178.35 (17)	C18—C19—C21—C22	−171.76 (18)

### Hydrogen-bond geometry (Å, °)

**Table:**

*D*—H···*A*	*D*—H	H···*A*	*D*···*A*	*D*—H···*A*
N1—H1A···N3^i^	0.86	2.38	3.1918 (18)	157
N1—H1B···N6^i^	0.86	2.35	3.2095 (18)	175
N4—H4C···N2^i^	0.86	2.29	3.1474 (19)	176
N4—H4B···N5^ii^	0.86	2.24	3.0834 (18)	166

Symmetry codes: (i) −*x*+2, −*y*, −*z*+1; (ii) −*x*+1, −*y*, −*z*+1.
